# Volatile Fatty Acids as Carbon Sources for Polyhydroxyalkanoates Production

**DOI:** 10.3390/polym13030321

**Published:** 2021-01-20

**Authors:** Karolina Szacherska, Piotr Oleskowicz-Popiel, Slawomir Ciesielski, Justyna Mozejko-Ciesielska

**Affiliations:** 1 Department of Microbiology and Mycology, Faculty of Biology and Biotechnology, University of Warmia and Mazury in Olsztyn, 10-719 Olsztyn, Poland; karolina.szacherska@uwm.edu.pl; 2 Water Supply and Bioeconomy Division, Faculty of Environmental Engineering and Energy, Poznan University of Technology, 60-965 Poznan, Poland; piotr.oleskowicz-popiel@put.poznan.pl; 3 Department of Environmental Biotechnology, University of Warmia and Mazury in Olsztyn, 10-719 Olsztyn, Poland; slawomir.ciesielski@uwm.edu.pl

**Keywords:** biodegradable polymers, bioproducts, polyhydroxyalkanoates, volatile fatty acids, waste stream

## Abstract

Waste of industrial origin produced from synthetic materials are a serious threat to the natural environment. The ending resources of fossil raw materials and increasingly restrictive legal standards for the management of plastic waste have led to research on the use of biopolymers, which, due to their properties, may be an ecological alternative to currently used petrochemical polymers. Polyhydroxyalkanoates (PHAs) have gained much attention in recent years as the next generation of environmentally friendly materials. Currently, a lot of research is being done to reduce the costs of the biological process of PHA synthesis, which is the main factor limiting the production of PHAs on the industrial scale. The volatile fatty acids (VFAs) produced by anaerobic digestion from organic industrial and food waste, and various types of wastewater could be suitable carbon sources for PHA production. Thus, reusing the organic waste, while reducing the future fossil fuel, originated from plastic waste. PHA production from VFAs seem to be a good approach since VFAs composition determines the constituents of PHAs polymer and is of great influence on its properties. In order to reduce the overall costs of PHA production to a more reasonable level, it will be necessary to design a bioprocess that maximizes VFAs production, which will be beneficial for the PHA synthesis. Additionally, a very important factor that affects the profitable production of PHAs from VFAs is the selection of a microbial producer that will effectively synthesize the desired bioproduct. PHA production from VFAs has gained significant interest since VFAs composition determines the constituents of PHA polymer. Thus far, the conversion of VFAs into PHAs using pure bacterial cultures has received little attention, and the majority of studies have used mixed microbial communities for this purpose. This review discusses the current state of knowledge on PHAs synthesized by microorganisms cultured on VFAs.

## 1. Introduction

Nowadays, petroleum-based plastics, due to their properties, are one of the most common materials in modern society. Constant production of materials based on synthetic polymers and their intensive human overconsumption leads to irreversible changes that have a negative impact on the natural environment and human health. The growing accumulation of non-biodegradable polymer wastes and the exhaustive reserves of fossil fuels prompted scientists to develop new biopolymer production technologies. Biopolymers are gaining more interest due to not only their biodegradability/compostability, but also biocompatibility, and bio-based nature. Various types of biodegradable polymers are being used in various fields. They are grouped depending on their composition. Polymers like starch and cellulose belong to the first group and are produced from agro-resources. The second group includes biopolymers synthesized by microorganisms during microbial processes, e.g., polyhydroxyalkanoates (PHAs). The third group consists of polymers that are conventionally and chemically synthesized (polylactic acid (PLA)). The fourth group contains blends of renewable resources with synthetic monomers (soy-based urethanes) [1]. However, until now, PLA and PHAs are the key players in the market for biodegradable polymers. PLA has gained much attention as the effective polymer for biomedical and packing composites. Nonetheless, due to fragility, reduced impact strength, and low thermal stability, PLA has become a hurdle for the application in engineering purposes [2]. Additionally, PHAs are attracting attention as the next generation of environmentally friendly materials and have become an alternative to synthetic polymers because they are completely biodegradable, biocompatible and can be produced from renewable sources [3]. The worldwide production of PHAs from commercial manufacturers reached up to 2.05 million tons in 2017 [4]. The interest in renewable, eco-friendly, and bio-based materials is still high, suggesting that the biopolymers market will be a serious player in the future. It is predicted that the global market value of PHA will grow from USD 57 million in 2019 to USD 98 million by 2024 [5].

PHAs are water-insoluble biopolymers synthesized by microorganisms and collected intracellularly as granular supplementary substances. Microorganisms accumulate PHAs as a reserve material, serving as energy in the presence of excess carbon source and limited concentration of essential nutrients (nitrogen, phosphorus, magnesium, and oxygen) [6]. Excess carbon is used for cell growth and the PHA storage process, and after depletion of carbon source, the stored polymers are used by microorganisms as a source of carbon and energy. PHAs are synthesized by gram-negative bacteria and gram-positive ones, which belong to genera such as *Pseudomonas*, *Bacillus*, *Cupriavidus*, *Aeromonas* and *Rhodobacter*. Thus far, only a few species of bacteria have met the optimal PHA production criteria. The most important are *Cupriavidus necator*, *Alcaligenes latus*, *Pseudomonas oleovorans* or *Pseudomonas putida*. Microorganisms produce a number of PHAs differing in chemical structure. These polymers consist of 100 to 30,000 monomers that bind to each other with ester bonds. The type of the biopolymer synthesized depends on the carbon source, the PHA producer and the bioprocess conditions. Over 150 different types of PHAs are synthesized by various bacterial species under different growth conditions. PHAs are classified based on the number of carbon atoms present in the biopolymer molecule and they can be divided into short-chain-length (PHA_SCL_), medium-chain-length (PHA_MCL_) and long-chain-length (PHA_LCL_) PHAs. PHA_SCL_ contains from three to five carbon atoms, e.g., poly-3-hydroxybutyric acid and poly-3-hydroxyvaleric acid. PHA_SCL_ is rigid and brittle thermoplastic biopolymer with a high degree of crystallinity (60%–80%) [7]. This type of PHA can be produced by a single species (e.g., *Cupriavidus necator*) as well as by mixed microbial cultures (MMC) derived from activated sludge. These polymers can be used in industry in the production of containers and packaging for everyday use. They can be the subject of investigation as food packaging materials due to their processing possibility to packaging films via thermoforming, a high water vapor barrier, a high barrier properties against CO_2_, a high oxygen barrier hampered the growth of aerobic microbes and the oxidative spoilage of unsaturated fatty acids [8]. The low oxygen transmission rate and water and CO_2_ barriers make PHAs an interesting basic materials for producing bottles for liquid foods, and also for CO_2_-containing liquids [9]. As a major drawback of using PHAs as food packaging material is still not satisfying a cost-performance scenario mainly because of the high expenses of raw materials [10]. PHA_MCL_ contain from 6 to 14 carbon atoms in the molecule, e.g., poly-3-hydroxyoctanoic acid or poly-3-hydroxydecanoic acid. They have elastomeric properties and are flexible biopolymers with low crystallinity, low tensile strength, high elongation to break, low melting point and glass transition (below room temperature). PHA_MCL_ are mainly produced by bacteria of the genus *Pseudomonas*. Due to their properties, these polymers can be widely used in the production of paints and pigments, as well as in medicine and pharmacy. PHA_LCL_ contain more than 14 carbon atoms in their structure, e.g., poly-3-hydroxyhexadecanoic acid [11]. This type of biopolyester is uncommon and knowledge about its properties and potential applications is still limited.

The main limitation in the production of PHA on a large scale is the high process costs, which are 15 times higher than in the case of conventional plastics [10]. Furthermore, the productivity of PHA during fermentations in terms of the amount of PHA formed per liquid volume per time (g PHA/L/h) is an important factor for techno-economical evaluation of this bioprocess. Therefore, it seems reasonable to use substrates that will not only be effectively used by microorganisms for growth and PHA synthesis, but can be obtained at prices that will guarantee the profitability of the process. These substrates could be waste materials such as organic industrial and food waste, and various types of wastewater [12,13,14]. The use of wastes for the production of biopolymers is a beneficial approach, ensuring the disposal of harmful substances and the creation of environmentally friendly materials [15]. Particular attention has been given to volatile fatty acids (VFAs), which are generated by anaerobic fermentation, such as acetic acid, propionic acid, butyric acid, valeric acid and caproic acid [16,17]. Previous results have shown that VFAs could be effectively used by microorganisms as a source of carbon and energy for the growth and production of PHAs [12,18].

For producing VFAs via anaerobic digestion, the organic fraction of municipal solid waste (OFMSW) is a promising potential substrate. The ever-growing amount of food waste in Europe, reaching about 90 million tons per year, requires the development of innovative solutions to the processing and disposing of it [19]. Therefore, OFMSW is subjected to anaerobic digestion to produce and extract VFAs, which can be further used as valuable sources of carbon for the production of value-added PHAs. Valentino et al. [20] conducted OFMSW fermentation for VFAs production at 55 °C using a 200 L continuous stirred-tank reactor (CSTR) with a hydraulic retention time (HRT) of 3.3 d and an average organic loading rate (OLR) of 20 kg VS/m^3^·d. An acidic environment (pH 5.0–5.6) was maintained by recirculation of digestate from a parallel thermophilic anaerobic fermenter. VFA production reached its maximum (22.3 g COD_VFA_/L) on day 9 (2.7 HRTs). In the fermented VFA stream, butyric acid (37.2%), acetic acid (23.4%), propionic acid (13.2%), valeric acid (10.9%) and caproic acid (9.6%) predominated. The authors also found smaller amounts of isobutyric acid (3.56%) and isovaleric acid (1.44%). Furthermore, it has been shown that pH can influence the rate of VFA production. Cheah et al. [21] revealed that a pH of nine can lead to a higher production of VFAs (high in acetic acid) than a pH of six. They demonstrated that the concentration of VFAs in wastewater increased up to 11.5 g/L.

The aim of this review is to present the challenges to bacterial PHA production using VFAs. Knowledge about the use of VFAs to synthesize PHAs is currently limited, so this review focuses on systematizing their potential in this field.

## 2. Characterization and Production of Volatile Fatty Acids

VFAs are linear short-chain fatty acids containing from two (acetic acid) to six (caproic acid) carbon atoms. Table 1 presents the characteristics and properties of VFAs.

Recently, special attention has been paid to the production and recovery of VFAs due to their high potential as renewable carbon sources and their broad range of applications [24]. VFAs can be used as sources in the production of biopolymers or biofuels (methane and hydrogen) and in the biological removal of nutrients from wastewater [25,26,27]. Due to the fact that VFAs contain a carboxylic group, they are also used in conventional organic chemistry as precursors of reduced chemical compounds and derivatives (aldehydes, alcohols, alkanes, ketones or esters) [28].

VFAs are intermediate products in the process of anaerobic digestion (AD) of various types of waste, such as food waste, municipal waste, agricultural waste, milk sewage, paper and cellulose sewage. Many microorganisms were reported to be capable of generating VFAs, such as acetic acid, propionic acid and butyric acid using a variety of substrates (Table 2). The AD process consists of a series of stages in which the organic matter is degraded by microorganisms under anaerobic conditions [29]. The synthesis of VFAs by mixed microbial cultures (MMC) is possible due to various metabolic pathways that occur inside cells, depending on the substrate used.

In many cases, co-fermentation of different wastes was employed, for example, agricultural waste and animal manure [30], or waste activated sludge and corn straw [31]. The purpose of co-fermentation is to balance the C/N ratio. Some experiments showed that an increase in the C/N ratio supported the growth of microorganisms responsible for acidogenesis [32,33]. In order to increase the efficiency of VFA production by MMCs, special attention should be paid to the conditions and parameters of the process. In particular, different hydraulic retention times (HRT), organic loading rates (OLR), temperatures and pHs can affect the productivity of VFAs. It is also possible that other by-products will be formed, e.g., long-chain fatty acids, alcohols, biohydrogen, biomethane, esters and other intermediates [34,35].

Bermúdez-Penabad et al. [36] studied the effect of different pH values (from 5.0 to 10.0) on the AD of tuna waste to produce VFAs. The authors showed that varying the pH influenced the production of VFAs. After 32 days of digestion of tuna waste, the highest total VFAs concentration at pH 8 was 30.611 mg COD/L. The resulting VFAs consisted mainly of acetic acid, propionic acid, butyric acid and valeric acid. Furthermore, acetic acid was the dominant product at all pH values tested. Den Boer et al. [37] used a co-culture consisting of *Klebsiella mobilis* and *Escherichia coli* to produce short-chain and medium-chain VFAs from kitchen biowaste and potato peels. The authors tested the effects of various feeding strategies to increase chain elongation under microaerobic conditions at pH 6.0–6.5. In the initial stages of the process, acetic acid and ethanol dominated, while in the later stages, an accumulation of propionic acid was observed, followed by accumulation of butyric and valeric acid. The highest level of final products, 325 mg/g TS, was obtained at pH 6.5, with a yield of 448 mg/L/h. Moretto et al. [38] optimized the fermentation process of urban organic waste to produce a stream rich in VFAs. The effects of different temperatures, pH values, HRTs and OLRs were studied in both batch and continuous processes. Using batch processes at a temperature of 37 °C and a pH of 9 ensured the production of VFAs using waste feedstock thermally pretreated for 76 h at 72 °C. At a later stage, these conditions were used in a continuous process, resulting in the efficient production of VFAs with high levels of VFAs (0.77 COD_VFA_/VS_(0)_ and 39 g COD_VFA_/L).

VFAs converted anaerobically from various waste streams could be a promising platform for PHA production. The process of PHA synthesis by microorganisms using waste-derived substrate required expensive enzymes, sterilization processes, and high energy demand, which increases the overall production cost. The synthesis of PHAs using low-cost materials, such as VFAs as the feedstock, is considered to be an economical approach. Furthermore, PHA production from VFAs seems to be a good approach since VFAs composition determines the constituents of PHA polymer and has a great influence on the properties of PHAs [39].

**Table 2:** Production of volatile fatty acids by microorganisms.

Volatile Fatty Acid	Bacteria	Substrate	Concentration (g/L)	Productivity (g/L/h)	References
Acetic acid	*Acetobacter aceti*	cheese whey	96.9	4.060	[40]
	*Clostridium acetium*	mixed gas (4% H_2_:18% Argon:78% CO)	1.3	nd	[41]
	*Clostridium lentocellum SG6*	paddy straw	30.9	nd	[42]
	*Moorella thermoacetica*	sugarcane straw hydrolysate	17.2	nd	[43]
	*Saccharomyces cerevisiae + Acetobacter pasteurianus*	glucose	66.0	0.367	[44]
Propionic acid	*Propionibacterium acidipropionici* (ATCC 4965)	lactate	15.1	0.113	[45]
		glycerol	6.8	0.051	
		sugarcane molasses	8.2	0.062	
	*Propionibacterium acidipropionici* (CGMCC 1.223)	glycerol	44.6	0.200	[46]
	*Propionibacterium acidipropionici* (ATCC 4875)	hemicellulose hydrolysate	18.0	nd	[47]
		chesse whey	19.7	0.980	[48]
	*Propionibacterium freudenreichii* CCTCC M207015	glucose	67.1	0.140	[49]
	*Propionibacterium freudenreichii* spp. *shermanii*	glycerol	9.0	0.180	[50]
Butyric acid	*Clostridium butyricum* S21	sucrose	20.0	0.210	[51]
	*Clostridium butyricum* ZJUCB	glucose	16.7	nd	[52]
	*Clostridium thermobutyricum* JW171K	glucose	18.4	2.400	[53]
	*Clostridium tyrobutyricum*	corn husk hydrolysate	20.8	0.420	[54]
		sugarcane bagasse hydrolysate	20.9	0.510	[55]

nd—not determine.

## 3. Synthesis of PHAs from VFAs by Pure Bacterial Cultures

**Figure 1:**
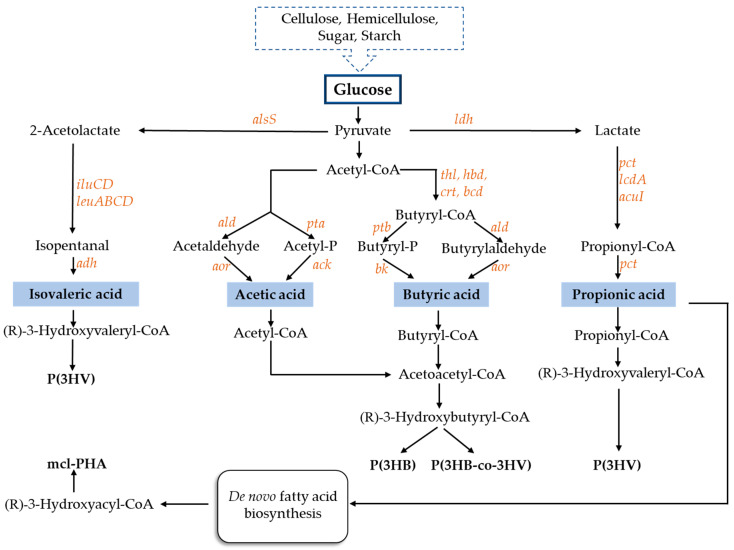
Scheme of metabolic pathways for volatile fatty acids production and polyhydroxyalkanoates synthesis. *ack*—acetate kinase, *acul*—acrylyl-CoA reductase, *adh*—alcohol dehydrogenase, *ald*—aldehyde dehydrogenase, *alsS*—acetolactate synthase, *aor*—aldehyde oxidoreductase, *bcd*—butyryl-CoA dehydrogenase, *bk*—butyrate kinase, *crt*—crotonase, *hbd*—hydroxybutyryl-CoA dehydrogenase, *ilvc*— keto-acid reductoisomerase, *ilvD*—dihydroxy acid dehydratase, *kivD*—2-keto acid decarboxylase, *lcdA*—lactoyl-CoA dehydratase, *ldh*—lactate dehydrogenase, *leuA*—2-isopropylmalate synthase, *leuB*—isopropylmalate dehydrogenase, *leuCD*—isopropylmalate isomerase complex, *pct*—propionyl-CoA transferase, *pta*—phosphotransacetylase, *ptb*—phosphotransbutyrylase, *thl*—thiolase.

In order to launch PHAs into the market, there is a need to reduce the costs involved in the production of these biopolymers. Therefore, the use of cheap substrates, including VFAs, may contribute to lowering the cost of obtaining the final bioproduct [56]. Numerous metabolic pathways are involved in VFA synthesis. VFAs are also the preferred carbon source for the synthesis of PHAs, because they are direct metabolic precursors in the PHA biosynthesis pathway (Figure 1). Several pure bacterial cultures are capable of producing PHAs in satisfactory amounts using VFAs as substrates (Table 3).

Poly(3-hydroxybutyrate) [(P(3HB)] is the most well-known and the most widely studied polyhydroxyalkanoic acid. It is a brittle biopolymer with low elasticity, high O_2_ barrier, good thermoplastic properties and poor mechanical properties [67]. Chakraborty et al. [57] investigated the ability of *Ralstonia eutropha* ATCC 17699 to produce PHAs using acetic, propionic and butyric acid. This strain synthesizes homopolymer P(3HB) at a concentration of about 31% of cell dry weight (CDW) using acetic and butyric acid. However, higher productivity (0.037 g PHA/g substrate) was observed in a cultivation supplemented with butyric acid. According to López et al. [18], *Methylocystis hirsuta* was able to produce up to 52% of the CDW when grown on acetic and butyric acid as co-substrates during artificial biogas-based cultivation under nitrogen limiting conditions. Higher P(3HB) content was revealed in *Bacillus megaterium* OU303A isolated from municipal sewage sludge [58]. The authors found that this strain synthesized 62.43% of CDW in the presence of propionic acid in a medium containing glycerol. A slightly lower concentration (58.63% of CDW) was observed when the growth medium was supplemented with glucose. The obtained data suggested that the addition of propionic acid supported PHAs synthesis. The bacteria accumulated higher amounts of PHAs when this VFA was added, as compared to the use of individual carbon sources (glucose and glycerol). However, a two times lower P(3HB) content was reached with *Bacillus* sp. INT005 cells cultured on butyric acid [64].

In addition to homopolymers, pure bacterial cultures are able to biosynthesize copolymers using VFAs as carbon sources. Studies have shown that incorporating other PHA monomers into P(3HB) will significantly improve the mechanical properties of a final product, reduce crystallinity and melting temperature, and increase flexibility [68]. Kumar et al. [69] reported that bacteria belonging to the *Bacillus* genus are capable of synthesizing copolymers during cultivations supplemented with a specific mixture of VFAs obtained through the controlled hydrolysis of various wastes, such as pea shells, apple pomace, onion peels, and potato peels. The authors found that *Bacillus thuringiensis* EGU45 and *Bacillus cereus* EGU43 cultured on pea shells hydrolysates accumulated P(3HB-co-3HV) copolymer with a 3HV content of 1% *w*/*w*. Furthermore, it was observed that the efficiency of PHA synthesis could be improved by the addition of onion peel hydrolysate together with the supplementation with glucose. Interestingly, VFAs were not detected after the PHA production phase, suggesting that the applied waste hydrolysates supported efficient bacterial growth, and biopolyester synthesis and accumulation. Furthermore, the effect of combining cultures on PHA production was evaluated by Munir and Jamil [59]. The authors confirmed that a co-culture of *Pseudomonas* sp. ST2 and *Bacillus* sp. CS8 was capable of producing up to 35% P(3HB-co-3HV) copolymer when grown on acetic and propionic acids in combination with glucose. When a glucose and propionic acid mixture was used as a feedstock, the individual strains produced more PHAs than with glucose as the only carbon source. Furthermore, it was observed that *Alcaligenes eutrophus* NCIMB 11599 produced a higher P(3HB-co-3HV) copolymer yield and 3HV fraction of the copolymer in a two-stage fed-batch cultivation when using valeric acid than when using propionic acid. Additionally, *Ralstonia eutropha* KCTC 2658 has been identified as a PHAs copolymer producer when cultured on a mixture of acetic, propionic, and butyric acid [61]. It was reported that P(3HB-co-3HV) production reached 50% CDW using 5 g/L of acetic-acid:propionic-acid:butyric-acid at a proportion of 1:2:2. Furthermore, the authors confirmed that the applied conditions enabled the production of biopolyesters with a 3HV molar fraction of 21 mol%. Interestingly, *Ralstonia eutropha* ATCC 17699 synthesized the copolymer with a higher 3HV fraction (35 mol%), but the cell dry weight and biopolymers content in cells were lower (1.2 g/L and 25% of CDW, respectively). In addition, it was observed that the proportion of VFAs to co-substrates has an influence on the biomass, PHAs content and biopolymer composition. According to Du et al. [63], a low ratio of propionic acid to glucose led to a low concentration of 3HV fraction, but high dry cell weight and P(3HB-co-3HV) productivity. In contrast, a high propionic-acid-to-glucose ratio led to a high 3HV concentration but a low P(3HB-co-3HV) content in *Ralstonia eutropha* cells.

In addition, Jeon et al. [70] determined that, by altering the levels of the acetyl-CoA pool, it is possible to improve the concentration of 3HV in P(3HB-co-3HV). They overexpressed acetyl-CoA acetyltransferase (*atoAD*) in a recombinant *Escherichia coli* YH090 carrying genes responsible for PHA synthesis (*bktB*, *phaB*, and *phaC*). Use of propionic acid as a carbon source stimulated copolymer synthesis, resulting in a 7.3-fold higher 3HV fraction (67.9 mol%) in the copolymer produced by the above-mentioned mutant than by the strain without the overexpression of *atoAD* genes. Further, *Hydrogenophaga pseudoflava* grown in a medium supplemented with non-hydrolyzed lactose whey and valeric acid was able to synthesize up to 40% P(3HB-co-3HV) with 5 mol% 3HV [71].

A substantial amount of P(3HB-co-3HV) copolymer (55% of CDW) was synthesized by *Cupriavidus necator* cultured on olive mill wastewater effluent rich in acetic, propionic and butyric acid (OMW_Acid_) without adding any exogenous substrate. OMW_Acid_ contained not only different short chain VFAs, but also polyphenols (1.2 g/L), N-NH4 (60 mg/L), proteins (1.56 g/L), and lipids (3.24 g/L). Furthermore, it was observed that OMW_Acid_ at concentrations of 75% and 100% inhibited the synthesis and accumulation of the copolymer. This work shows that residues from the fermentation of wastes can be used for the biosynthesis of bacterial biopolymers [62]. Such approaches can potentially reduce the production costs of PHAs and offer environmental benefits through the reuse of wastes. It was demonstrated that the type II methanotroph *Methylocystis hirsuta* is capable of synthesizing P(3HB-co-3HV) copolymer using artificial biogas with the addition of VFAs [18]. The highest copolymer concentration was observed in a cultivation with valeric acid as a co-substrate (53.8% of CDW), and the highest 3-hydroxyvalerate content (13.5%) was found within the biocomposite. The same strain accumulated trace amounts of these biopolymers using pure VFAs as the only carbon sources (from 1.1 to 9.0% of CDW). Recently, Ferre-Guell and Winterburn [60] obtained increased P(3HB-co-3HV) copolymer concentrations in a cultivation of *Haloferax mediterranei* grown on a mixture of butyrate and valerate acids with the addition of surfactants to increase substrate bioavailability. The studied strain was able to synthesize up to 59% of the copolymer, resulting in a productivity rate of 10.2 mg/L/h in the fed-batch fermentation. These results demonstrate that a VFA mixture with the addition of Tween 80 supports the synthesis of polymers containing the desired 3HV fraction of 43 mol%.

Thus far, only one study reported the production of mcl-PHAs using VFAs. Cerrone et al. [66] studied the effect of nitrogen limitation on the growth, and synthesis and accumulation of mcl-PHAs by three *Pseudomonas putida* strains (KT2440, CA-3 and GO16) using VFAs produced from the anaerobic digestion of a lignocellulosic substrate. The effluent that was used as a carbon source to support growth and mcl-PHA synthesis consisted of 15.3 g/L of VFAs (from acetic acid to valeric acid) with a predominance of butyric acid (12.8 g/L). The results revealed that the analyzed strains differed in their ability to grow and accumulate mcl-PHAs. The highest mcl-PHA concentration (44% CDW) was detected in the cells of *Pseudomonas putida* KT2440 cultured on butyric acid and acetic acid. Similar levels of biopolyesters were extracted from *Pseudomonas putida* CA-3 grown on acetic, propionic or butyric acid. Furthermore, the production of mcl-PHAs was lower with the GO16 strain than with the KT2440 and CA-3 strains, regardless of the type of VFA. However, gas chromatography analysis revealed that the monomeric composition of the mcl-PHAs extracted from *P. putida* KT2440 and CA-3 cells was similar. The authors reported that 3-hydroxydecanoic acid (3-HD) was the major component. Furthermore, this biopolyester was found to contain lesser amounts of 3-hydroxyoctanoic acid (3-HO) and 3-hydroxydodecanoic acid (3-HDD), and trace amounts of 3-hydroxytetradecanoic acid (3-HTD). Interestingly, *Pseudomonas putida* GO16 produced mcl-PHAs containing higher levels of 3-HDD monomer than those produced by the KT2440 and CA-3 strains, suggesting that its level is dependent on bacterial strain.

## 4. Synthesis of PHAs from VFAs by Mixed Microbial Cultures

Recently, interest in using MMC for the production of PHAs has increased significantly among researchers. MMC are communities of microorganisms that have the ability to cooperate with each other by carrying out specific intracellular and extracellular reactions. The process of using MMC for the synthesis of PHAs can be economically competitive with the production of PHAs by pure bacterial cultures [72]. Using mixed cultures is simpler and requires less investment and operational expenses in terms of sterile conditions and a feedstock preparation. VFAs such as acetate, propionate, butyrate and valerate can be used efficiently by mixed microbial cultures for the synthesis of PHAs (Table 4).

Acetic acid is one of the best-studied carbon sources for the synthesis of PHAs by mixed cultures under feast and famine conditions. In the metabolic pathway, acetic acid is transformed into P(3HB). The efficiency and rate of polymer synthesis depend on the operating conditions of the bioreactor. Serafim et al. [73] found that aerobic dynamic substrate feeding (ADF) in a sequencing batch reactor (SBR) operated under ammonia limitation favored the selection of cultures with a high P(3HB) storage capacity. Furthermore, the authors observed that the P(3HB) specific storage rate could have been inhibited due to the acetic acid concentration. They confirmed that P(3HB) content reached 67.5% when acetic acid was supplied in one pulse at a concentration of 180 Cmmol/L. However, feeding the substrate pulse-wise caused an increase in the P(3HB) storage rate (78.5%). Beun et al. [74] reported that, during the feast period in an SBR reactor, the P(3HB) content reached only 40% CDW. It was also shown that, in the subsequent fed-batch, the enriched mixed culture was able to produce up to 89% CDW within 7.6 h of continuous feeding with acetic acid [75]. Furthermore, it was concluded that, the longer the cultivation in the feast-famine SBR, the higher the P(3HB) production rate. The average specific rate of PHB production over the first 7.6 h was 1.4 C-mol/C-mol/h (1.2 g/g/h). Moreover, it was proved that the presence of acetic acid stimulates the consumption of butyrate and propionate due to the reactivation of acetyl-CoA, and thus supports the internal activity of cellular metabolism. Thus, it can be assumed that acetic acid is one of the most appreciated VFAs in the production of PHAs by microorganisms [76].

**Table 4:** Production of PHAs by mixed microbial cultures using volatile fatty acids.

Carbon Source	Type of PHA	PHA (%)	References
Acetic acid	P(3HB)	40.0	[74]
	P(3HB)	78.5	[73]
	P(3HB)	89.0	[75]
Municipal wastewater + acetic acid	P(3HB)	30.0	[77]
Fermented paper mill wastewater (acetic acid, propionic acid, butyric acid, valeric acid)	P(3HB-co-3HV)	48.0	[78]
Fermented molasses (acetic acid, propionic acid, butyric acid, valeric acid)	P(3HB-co-3HV)	66.0	[14]
Fermented food waste (acetic acid, propionic acid, butyric acid, valeric acid) + dewatered sludge	P(3HB-co-3HV)	64.5	[79]
Fermented paperboard mill wastewater (acetic acid, propionic acid, butyric acid, valeric acid)	P(3HB-co-3HV)	67.4	[80]
Sludge hydrolysis liquid (acetic acid, propionic acid, butyric acid, valeric acid)	P(3HB-co-3HV)	24.1	[81]
Fermented crude glycerol	P(3HB-co-3HV)	76.0	[82]
Fermented wood waste (acetic acid, propionic acid, butyric acid)	P(3HB-co-3HV)	50.3	[83]
Fermented cheese whey (acetic acid, propionic acid, butyric acid, valeric acid)	P(3HB-co-3HV)	30.0	[84]

The substrate composition seems to be an important factor that strongly affects the diversity of PHA monomers. Fradinho et al. [76] evaluated the feeding of an acetate-enriched photosynthetic mixed culture (PMC) with mixed VFAs. The authors showed that acetic acid and butyric acid supported P(3HB) homopolymer synthesis, whereas propionic acid supported the formation of P(3HB-co-3HV) copolymers consisting of 51% 3HV. Furthermore, a low acetate concentration (<30 CmM) and specific light intensities around 20 W/gX were determined to be the optimal operating conditions that enable the improvement of P(3HB-co-3HV) content from 15% to 30% in less than 4 h [74]. Additionally, Lemos et al. [85] confirmed that, by manipulating the feed VFA profiles, polymers with different monomer compositions could be synthesized. The results indicated that sludge fed with acetate was able to produce a P(3HB) homopolymer, whereas sludge fed with propionate was capable of synthesizing a P(3HB-co-3HV) copolymer. Furthermore, switching the substrate feeds, i.e., feeding propionic acid to sludge adapted to acetate and acetic acid to sludge adapted to propionate, led to the production of a P(3HB-co-3HV-3HMV) terpolymer.

In the field of PHA biosynthesis, attention has focused on the possibility of using pretreated sludge as a possible carbon source for the synthesis of these polymers. Liao et al. [81] demonstrated that VFAs from heat-pretreated sludge hydrolyzed liquid could support PHA synthesis and accumulation. The authors observed that different heat pretreatment temperatures affect the composition of VFAs, and consequently, the biopolymer productivity rate. Hydrolyzed liquid that was heat pretreated at 60 °C was the optimal carbon source for P(3HB-co-3HV) synthesis, and the maximal copolymer content was 24.1% CDW. At that temperature, acetic acid was the predominant VFA in the hydrolyzed liquid, whereas propionic, butyric and valeric acid were present in smaller amounts. Chua et al. [77] noted that sludge acclimatized with municipal wastewater supplemented with acetic acid could accumulate PHAs up to 30% CDW, whereas sludge without this VFA accumulated 20% CDW.

Wastewater from waste-glycerol fermentation supplemented with different amounts of acetic and propionic acids improves PHA production capacity. In an SBR reactor, a mixed microbial community fed a substrate of organic acids supplemented with acetic acid produced P(3HB), while propionic acid supported P(3HB-co-3HV) synthesis. Genetic analysis revealed that *Thauera* sp. predominated in the reactor with acetate, while *Paracoccus denitrificans* predominated in the reactor with propionate [86]. Additionally, Burniol-Figols et al. [82], using fermented crude glycerol and mixed microbial consortia, confirmed the possibility of selectively converting VFAs into PHAs. The authors extracted up to 76% P(3HB-co-HV) from an SBR reactor where the dominant bacterial taxa were *Amaricoccus* and *Thauera*, which accounted for 56.3%–72.4% of the whole microbial community.

Chen et al. [79] showed the effect of VFAs obtained from co-fermentation of municipal wastewater and food waste on the production of PHAs, and revealed that the feeding regimes affect the rate of PHA synthesis. Continuous pulsed feeding with fermentation fluid containing VFAs (acetic acid, propionic acid, butyric acid, and valeric acid) was most effective, leading to the highest P(3HB-co-3HV) synthesis rate of 64.5%. A similar copolymer content (66% of CDW) was observed when mixed cultures were supplemented with VFAs from fermented molasses [14]. The use of a continuous feeding strategy seems to be more efficient than a pulse feeding strategy and results in higher biopolymer productivity. Furthermore, a continuous feeding regime increased the 3HV content by 8% compared to one using pulse-wise feeding. Lower copolymer content (50.3% of CDW) was observed by Li et al. [83] when VFAs obtained from the co-fermentation of pretreated wood waste and sewage were used as a feedstock. The authors showed that the PHA production rate reached 0.237 g COD PHA/L/h. Monomeric composition analysis revealed that mixed microbial consortia were able to produce P(3HB-co-3HV) copolymer that contained only the 6 mol% 3HV fraction. Promising results were described by Bengtsson et al. [78], who demonstrated that activated sludge cultivated on VFAs produced by acidogenic fermentation of paper mill wastewater was capable of synthesizing 48% CDW of P(3HB-co-3HV). A higher PHA content was obtained using acetate-enriched bacteria growing on the same fermented effluent [80]. The final biopolymer content was 58.57%, while the PHA yield reached 0.46 g PHA/g VFA. High throughput sequencing of the bacterial *16S* rRNA gene confirmed that *Proteobacteria* and *Bacteroidetes* spp. increased during the enrichment and accumulation phases from 37.4% to 77.6% and from 2.49% to 17.66%, respectively. In the production of PHAs from waste streams, the presence of salts and compounds that inhibit biopolymer productivity should be taken into account. Palmeiro-Sánchez et al. [87] showed that the concentration of sodium chloride has an effect on biopolymer synthesis and accumulation. A mixed microbial culture enriched on a mixture of VFAs containing sodium chloride produced PHAs less efficiently than a culture without sodium chloride (53% of CDW).

## 5. Value Chain for Deriving Polyhydroxyalkanoates from VFAs

Among many different biodegradable polymers, PHAs deserve special attention due to their properties, which are similar to those of conventional plastics, as well as the fact that they are completely biodegradable to water and carbon dioxide in different environmental conditions [88]. Many efforts have been made in the past decades to increase PHA performance on both the laboratory and pilot levels. The main challenge in the commercialization of PHAs is the high production cost, which results from the low efficiency of the microbial processes.

**Figure 2:**
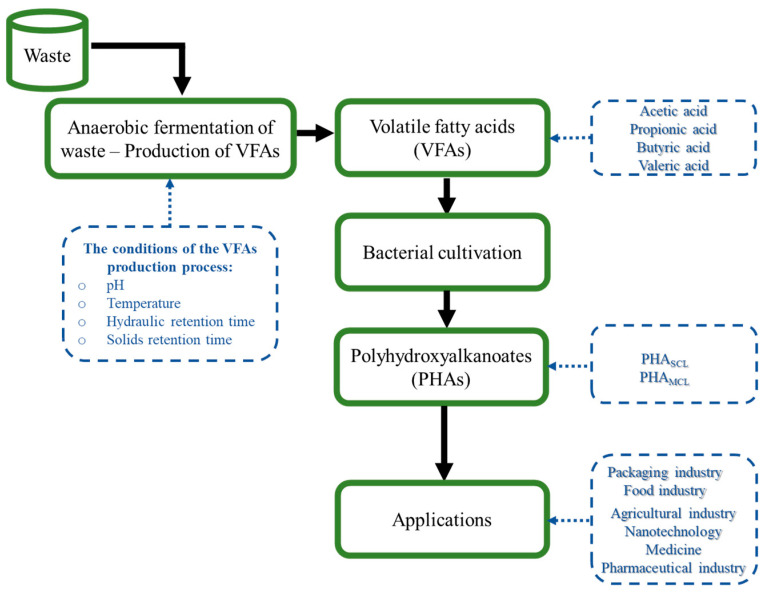
PHAs process value chain using volatile fatty acids.

PHAs derived from microbial processes are much more expensive than those produced using petrochemical processes. The cost of producing disposable products with P(3HB) is higher (7–10 EUR/kg) compared to production with synthetic polymers [89]. The final production costs of PHAs depend mainly on the price of the substrates used as carbon sources for microbial growth. Ensuring that the optimal carbon source is present in the microbial growth medium usually accounts for about 50% of the total cost of the PHA production process, which is why low-cost waste materials that can be effectively used to accumulate PHAs are constantly sought [90]. The conversion of waste materials to PHAs seems to be a solution to make the production of these biopolymers feasible (Figure 2) [91].

Many reports show that VFAs could be good carbon sources for PHA synthesis. However, most studies on PHA synthesis are conducted using petrochemical-derived VFAs as carbon sources. In order to reduce the overall costs of PHA production to a more reasonable level, it will be necessary to design a bioprocess that maximizes VFA production, which will be beneficial for the PHA synthesis. While the generation of large amounts of VFAs is desired, it is also necessary to prevent the toxic effect of VFAs on bacterial cells [92]. Mixed-culture PHA production offers the possibility of using renewable VFAs from wastes and industrial effluents. Utilizing fermented wastes or waste sludge as useful resources for VFA production is a promising alternative for reducing PHA production costs.

Additionally, a very important factor that affects the profitable production of PHAs from VFAs is the selection of a microbial producer that will effectively synthesize the desired bioproduct. In recent years, many microorganisms with the ability to synthesize various types of PHA have been studied. The PHA productivity of a microorganism is dependent on many factors, such as the type of carbon source, the temperature, the pH and the concentration of macro and microelements. For PHA production on a large scale, microorganisms should be able to utilize wastes as carbon sources, achieve a good growth rate and produce large amounts of PHAs. However, the concentration of fatty acids may result in toxicity for bacterial cells, that, up to a point, can be overcome by the fast growth of cells [93]. To enhance PHA productivity using VFAs, efforts can be made to use genetically recombined microorganisms. Furthermore, different types of PHAs are suitable for different applications. In particular, mcl-PHAs have gained much attention in recent years. Due to their thermoplasticity and biocompatibility, they can be used for medical or pharmaceutical applications. Thus far, only one study on the biosynthesis of mcl-PHAs has focused on substrates such as VFAs [66]. After bacterial cultivation, the downstream processing of PHA plays a crucial role in the production process. The separation of PHAs from non-PHA cell mass and their purification is technically challenging, especially considering the PHAs extraction that are formed in a wastewater treatment facility. Some downstream processing strategies have recently been comprehensively discussed by Surendran et al. [94].

Many companies have tried to increase PHA productivity and to commercialize them on the market. Currently, the bacterial polymer market is small. Scientists are making every effort to optimize the production process, e.g., by using low-cost and waste materials. To successfully implement the widespread commercial production of PHAs, the culture conditions, the microbial growth conditions and the method of PHA extraction will need to be taken into account.

## 6. Challenges and Future Perspectives

Biopolymers have received considerable attention in recent years as consumer preferences are shifting toward the use of biodegradable commodities. Among the various types of bio-based polymers, PHAs seem to be the next generation of environmentally friendly materials since their global market size is expected to considerably grow. Due to their useful properties, they are one of the most investigated class of biopolymers estimated to replace some of the today’s synthetic polymers. However, the major limitation for PHAs wide industrialization is the high overall cost of their production, mainly because of the price of the carbon substrate used in the microbial cultivation process. Therefore, there is still a need to find efficient substrates to maintain economic feasibility of PHAs commercialization. VFAs are promising carbon sources for PHA production. So far, the conversion of VFAs into PHAs using pure bacterial cultures has received little attention, and the majority of studies have used MMC for this purpose. Indeed, the production of PHAs by MMCs capable of effectively accumulating these polymers can be a cost-effective alternative to their production by pure bacterial cultures, which require sterile process conditions. PHA fermentation using MMC can be continuously conducted in unsterile conditions. Despite this, cultivation with a pure culture is often preferred because it usually leads to reproducible results.

**Figure 3:**
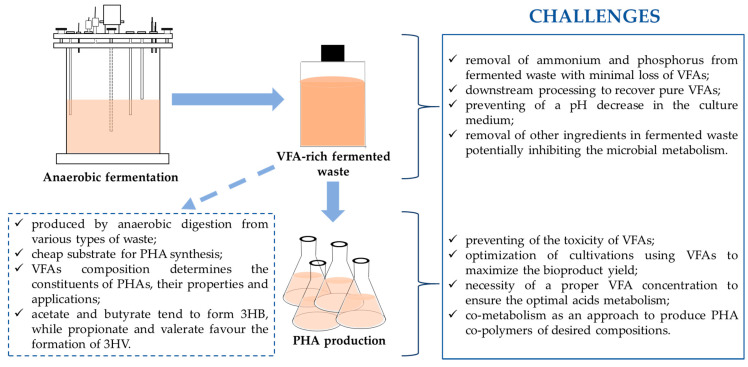
Challenges in producing polyhydroxyalkanoate biopolymers using VFAs.

To make PHA production a reality through fermentation using VFAs, there is a need to focus on a certain area, as discussed here. Additional investigations are needed to overcome some difficulties in the use of VFAs for producing PHAs. There are a number of challenges in microbial PHA synthesis from VFAs (Figure 3).

One of the main challenges is the regulation of ammonium and phosphorus in the VFA-rich fermented waste. It is known that the excess of these nutrients would promote the growth of microorganisms and reduce the PHA yield. The limited nitrogen and phosphorus conditions are of major importance for the effectiveness of the PHA synthesis process [95]. Struvite precipitation was proved to be effective in the rapid removal of these components from fermented waste with a minimal loss of VFAs [96]. Furthermore, the fermented waste should be filtered, especially in the case of PHA production by pure microbial culture [97]. Moreover, one major drawback is the toxicity of VFAs that could inhibit bacteria growth rates; therefore, optimization of the fermentation process using VFAs towards PHA production is crucial to maximize the bioproduct yield [98]. It seems to be necessary to know a proper concentration of VFAs which ensures the optimal acids metabolism to support a bacterial growth to a maximum level and high PHA productivity. Moreover, it was determined that the optimum level of VFAs available to bacteria increases the undissociated VFAs concentration inside their cytoplasm. The higher concentration of VFAs the higher dissociation that influences on the cytoplasm acidity. The reduction in a proton gradient across the membrane has an impact on the rising osmotic pressure that reduces the VFAs uptake inhibiting not only cell activity but also PHAs synthesis and accumulation. VFAs in undissociated form can penetrate the bacterial cell membrane and dissociate inside the cell, which may contribute to blocking ATP synthesis and disturbing the physiological balance [99]. Therefore, some energy must be used to restore the physiological balance in the cell, which contributes to the reduction of energy used for the growth and accumulation of PHAs by microorganisms. However, if the dissociated form of VFAs is present in high concentrations in the fermentation system then can cause an increase in ionic strength and in a consequence cell lysis [39]. Furthermore, VFAs may aggravate a decrease of pH in the culture medium and in a consequence may cause the overall bioprocess failure. In addition, VFAs-rich fermented waste may also contain ethanol, which can lead to high permeability of the cell membrane as well as suppressing the metabolism [100].

It is known that the chain-length of the VFA influences on the composition, properties and application of the synthesized PHAs. The data suggested that the feeding of mixed microbial culture with acetic and butyric acids results in the production of 3HB whereas propionic and valeric acids favor the synthesis and accumulation of 3HV [95]. Due to the poor properties, P(3HB) has limited applications; therefore, the production of PHA co-polymers with better properties is a good approach to overcome the limitations of P(3HB). By the appropriate combination of substrates and supplements, it is possible to produce PHA co-polymers of desired compositions. PHA co-polymers can be achieved by supplementing the feed with volatile fatty acids or through hydrolysates of different biowastes [101,102]. Some strategies of co-utilization were described above (Table 3).

Extensive research and development are still needed to optimize the synthesis of PHAs using VFAs. Only few microbes have been explored for their potential to produce PHAs using VFAs. Isolation and identification of new microbial cultures for a higher productivity is required. The PHAs productivity using VFAs generated in the process of anaerobic digestion of various types of waste is reported to be still too low. The major bottleneck is a downstream processing. There is a need to focus the attention in the area to recover VFAs with a higher productivity and purity. VFAs could also hamper the growth and PHAs synthesis by bacteria. Microbe engineering to tolerate VFAs as feedstocks may be an appropriate approach. Moreover, the great efforts should be made to separate VFAs from a fermentation broth using the cost-efficient method to ensure that the process of PHAs production will be economically feasible.

Advances in bioinformatics and metabolic engineering, along with expanding knowledge of microbial genomes may open many opportunities for the introduction of new metabolic pathways and modification of these pathways in pure bacterial cultures. A better understanding of genetic and metabolic regulation could lead to optimization of PHA production processes and the efficient utilization of VFAs as a source of carbon. In addition, the use of genetically modified microorganisms should be investigated as a potential method of improving PHAs production in the bioprocesses using VFAs. From a practical point of view, it is essential to construct a bacterial platform that will provide a consistent PHA molecular structure, thus ensuring stable material properties, which are essential for further applications of these polymers.

## Figures and Tables

**Table 1:** Properties of volatile fatty acids [22,23].

Volatile Fatty Acid	Boiling Point (°C)	Solubility (g/dm^3^)	COD_equivalent_ (g COD/g)	Vapor Pressure (mm Hg)	Smell
Acetic acid	117	Complete	1.066	20.9	Sharp smell of vinegar
Propionic acid	141	Complete	1.512	3.3	Sharp, rancid, acrid
Butyric acid	164	Complete	1.816	1.65	Rancid butter, sweat
Isovaleric acid	177	20	2.037	1	Rancid fat, blue cheese
Valeric acid	186	40	2.037	1	Sweat, valerian
Caproic acid	206	11	2.204	0.2	Sharp, sour

**Table 3:** Production of PHAs by pure bacterial cultures using volatile fatty acids.

Bacteria	Type of PHA	Carbon Source	Biomass (g/L)	PHA Content (%)	PHA Productivity (g/L/h)	References
*Ralstonia eutropha* ATCC 17699	P(3HB)	acetic acid	5.4	30.8	0.013	[57]
		propionic acid	14.0	29.3	0.036	
		butyric acid	14.5	31.9	0.037	
*Bacillus megaterium* OU303A	P(3HB)	propionic acid + glucose	nd	62.4	nd	[58]
		propionic acid + glycerol	nd	57.2	nd	
*Pseudomonas* sp. ST2	P(3HB-co-3HV)	propionic acid + glucose	nd	34.0	nd	[59]
*Bacillus* sp. CS8		propionic acid + glucose	nd	24.0	nd	
*Pseudomonas sp.* ST2 + *Bacillus* sp. CS8		acetic acid+ propionic acid + glucose	nd	35.0	nd	
*Haloferax mediterranei*	P(3HB-co-3HV)	butyric acid + valeric acid + Tween 20	nd	58.9	0.010	[60]
*Ralstonia eutropha* ATCC 17699	P(3HB-co-3HV)	acetic acid + propionic acid + butyric acid	1.2	25.0	nd	[61]
*Ralstonia eutropha* KCTC 2658	P(3HB-co-3HV)	acetic acid + propionic acid + butyric acid	1.5	50.0	nd	
*Cupriavidus necator*	P(3HB-co-3HV)	olive mill wastewater effluent rich in acetic, propionic and butyric acid	2.0	55.0	0.022	[62]
*Methylocystis hirsuta* DSM 18500	P(3HB-co-3HV)	acetic acid	nd	2.4	nd	[18]
		propionic acid	nd	1.1	nd	
		butyric acid	nd	1.8	nd	
		valeric acid	nd	9.0	nd	
	P(3HB)	acetic acid + biogas	nd	52.3	nd	
	P(3HB-co-3HV)	propionic acid + biogas	nd	47.9	nd	
	P(3HB)	butyric acid + biogas	nd	52.2	nd	
	P(3HB-co-3HV)	valeric acid + biogas	nd	53.8	nd	
*Ralstonia eutropha* DSM 545	P(3HB-co-3HV)	glucose + propionic acid	52.1	78.3	0.74	[63]
*Bacillus* sp. INT005	P(3HB)	butyric acid	0.8	31.5	nd	[64]
	P(3HB-co-3HV)	valeric acid	0.7	18.8	nd	
*Corynebacterium hydrocarboxydans*	P(3HB-co-3HV)	acetic acid	nd	21.0	nd	[65]
*Nocardia lucida*	P(3HB-co-3HV)	acetic acid	nd	20.0	nd	
*Rhodococcus* sp.	P(3HB-co-3HV)	acetic acid	nd	29.0	nd	
	P(3HB-co-3HV)	valeric acid	nd	43.0	nd	
*Pseudomonas putida* KT2440	mcl-PHA	acetic acid	~0.5	26.0	nd	[66]
		propionic acid	~0.5	29.0	nd	
		butyric acid	~0.5	44.0	nd	
		valeric acid	~0.5	19.0	nd	
*Pseudomonas putida* CA-3		acetic acid	~0.6	~40.0	nd	
		propionic acid	~0.6	~40.0	nd	
		butyric acid	0.7	~40.0	nd	
		valeric acid	~0.6	24.0	nd	
*Pseudomonas putida* GO16		acetic acid	0.4	~10.0	nd	
		propionic acid	0.1	~10.0	nd	
		butyric acid	0.8	28.0	nd	
		valeric acid	0.2	~10.0	nd	

nd—not determined.

## Data Availability

Not applicable.
